# A Mental Timeline for Duration From the Age of 5 Years Old

**DOI:** 10.3389/fpsyg.2018.01155

**Published:** 2018-07-10

**Authors:** Jennifer T. Coull, Katherine A. Johnson, Sylvie Droit-Volet

**Affiliations:** ^1^Aix-Marseille University, CNRS, LNC (UMR 7291), Marseille, France; ^2^School of Psychological Sciences, University of Melbourne, Melbourne, VIC, Australia; ^3^CNRS, Laboratoire de Psychologie Sociale and Cognitive, UMR 6024, Université Clermont Auvergne, Clermont-Ferrand, France

**Keywords:** timing, time perception, duration, space, position, development, timeline, magnitude

## Abstract

Both time and number can be represented in spatial terms. While their representation in terms of spatial magnitude (distance or size) might be innate, their representation in terms of spatial position (left/right or up/down) is acquired. In Western culture, the mental timeline represents past/future events or short/long duration on the left/right sides of space, respectively. We conducted two developmental studies to pinpoint the age at which the mental timeline for duration begins to be acquired. Children (aged 5–6, 8, or 10 years old) and adults performed temporal bisection tasks in which relative spatial position (left/right) was manipulated by either arrow direction (Experiment 1) and/or lateralized stimulus location (Experiments 1 and 2). Results first confirmed previous findings that the symbolic representation of spatial position conveyed by arrow stimuli influences the perception of duration in older children. Both 8 and 10 year olds judged the duration of leftward arrows to be shorter than that of rightward arrows. We also showed for the first time that as long as position is manipulated in a non-symbolic way by the visual eccentricity of the stimuli, then even 5–6 year olds’ perception of duration is influenced by spatial position. These children judged the duration of left-lateralized stimuli to be shorter than that of either right-lateralized or centrally located stimuli. These data are consistent with the use of a mental timeline for stimulus duration from the age of 5 years old, with short duration being represented on the left side of space and long duration on the right. Nevertheless, the way in which left and right were manipulated determined the age at which spatial position influenced duration judgment: physical spatial location influenced duration perception from the age of 5 years old whereas arrow direction influenced it from the age of 8. This age-related dissociation may reflect distinct developmental trajectories of automatic versus voluntary spatial attentional mechanisms and, more generally highlights the importance of accounting for attentional ability when interpreting results of duration judgment tasks.

## Introduction

Time is often represented in spatial terms. For example, “we waited a *long* time” or “take a look *back* over your career." Indeed, the notion that the perception of time is merely a subjective construct derived from the movement of objects through space has been around for decades. James (1890) drew an analogy between the perception of time and space, stating “date in time corresponds to position in space” (p. 610). Piaget (1969) proposed that “time and space form an inseparable whole” (p. 1) and demonstrated that young children cannot disentangle the notions of time and space, with long duration being equated to long distance. Indeed, duration is represented in terms of spatial distance not only in adults and children (Casasanto et al., 2010; Charras et al., 2017), but also even in neonates (de Hevia et al., 2014) and monkeys (Merritt et al., 2010; Mendez et al., 2011).

This spatialization of time has been encompassed into two major theoretical frameworks, in which both time and number are represented in spatial terms. On the one hand, A Theory of Magnitude (ATOM) suggests that the dimensions of space (i.e., size), time (i.e., duration), and number (i.e., quantity) are all processed by a single, innate magnitude processing system (Walsh, 2003). Within this framework, small size is associated with short duration (and low quantity) and large size with long duration (and large quantity). On the other hand, the mental timeline (Bonato et al., 2012; Bender and Beller, 2014; Magnani and Musetti, 2017) or mental numberline (Hubbard et al., 2005) theories suggest that time and number are ordered along a linear spatial axis. As opposed to ATOM, this representation is thought to be culturally acquired rather than innate: in Western cultures, the left side of space is associated with short duration (Vallesi et al., 2008; Vicario et al., 2008) or early/past times (Santiago et al., 2007) while the direction is reversed (Fuhrman and Boroditsky, 2010; Ouellet et al., 2010) or rotated to the vertical axis (Boroditsky et al., 2011) in other cultures. The mental timeline also operates in the frontal (front-back) axis in adults (Torralbo et al., 2006; Ulrich et al., 2012; Eikmeier et al., 2013; de la Fuente et al., 2014) and children (Charras et al., 2017), and this egocentric representation of time may influence the way we conceptualize time even more strongly than horizontal (left–right) or vertical (up–down) orientations (Eikmeier et al., 2015a).

The mental timeline and ATOM are not mutually exclusive theories. Rather, they are based on complementary ways of measuring space (see also Núñez and Cooperrider, 2013; Winter et al., 2015). While ATOM emphasizes spatial *magnitude* (how big something is), the mental timeline emphasizes spatial *position* (where something is). To put it another way, ATOM is framed more in terms of coordinate spatial relations (i.e., metrical distance, which allows for measures of magnitude such as short/long) while the mental timeline depends upon categorical spatial relations (which allows for measures of relative position, such as left/right or above/below). Converging behavioral and neuroscientific data underline the distinction between these two forms of spatial measurement (Kosslyn et al., 1989), with categorical processing recruiting left parietal cortex and coordinate processing recruiting the right (Jager and Postma, 2003; Kranjec and Chatterjee, 2010). Moreover, spatial neglect patients with right hemisphere damage underestimate stimulus duration (Oliveri et al., 2009; Magnani et al., 2011), consistent with a role for the right hemisphere in magnitude processing. On the other hand, patients with left hemisphere lesions failed to show the usual effects of leftward prismatic adaptation on timing performance (Magnani et al., 2011), consistent with a role for the left hemisphere in processing relative position. In summary, while both ATOM and the mental timeline theory advocate a spatial representation of time, they conceptualize the nature of the spatial representation in different terms.

Time can also be conceptualized in terms of either magnitude or relative position. Duration refers to the length of time an event lasts (its temporal magnitude) while order refers to the moment in time at which an event occurs (its relative temporal position). The mental timeline has been suggested to represent both of these measures of time (Bonato et al., 2012). In terms of temporal *position*, stimuli representing events in the past (or future) are processed more quickly when they appear in the left (or right) side of space (Santiago et al., 2007) or when they are responded to with left (or right) response keys (Weger and Pratt, 2008). In terms of temporal *magnitude*, duration is under (or over)-estimated for stimuli representing the left (or right) side of space (Vicario et al., 2008; Droit-Volet and Coull, 2015) or when attention was shifted to the left (or right) side of space by optokinetic stimulation (Vicario et al., 2007) or prismatic adaptation (Frassinetti et al., 2009). Similarly, reaction times are faster when short- (or long-) duration stimuli are responded to with the left (or right) hand (Conson et al., 2008; Ishihara et al., 2008; Vallesi et al., 2008) or are paired with left (or right) sided primes (Di Bono et al., 2012). This spatial influence even transcends sensory modality, with visual stimuli being under- (or over-) estimated when auditory distractors were presented to the left (or right) ear (Vicario et al., 2009).

As mentioned previously, the mental timeline is acquired through culture and/or experience (Bonato et al., 2012; Casasanto and Bottini, 2014; Magnani and Musetti, 2017). It depends heavily on reading experience, and can be observed in blind, Braille-reading participants, as well as in sighted individuals (Bottini et al., 2015). However, the developmental trajectory of this spatial representation of time appears to differ as a function of the temporal measurement in question (duration or order). For example, Tillman et al. (2017) very recently showed that during language development, children aged 4–6 make use of a left–right mental timeline to convey the meaning of words that refer to relative temporal position (deictic time words, such as “yesterday” or “next year”). By contrast, children’s understanding of the relative remoteness of these words from the present time did not develop until age 7. These data suggest that the mental timeline is first used to conceptualize the temporal order of events while its use in representing their temporal magnitude emerges later in development. Consistent with this idea, Droit-Volet and Coull (2015) found that the presentation duration (i.e., temporal magnitude) of rightward-facing arrows was overestimated (and leftward ones underestimated) in 10 year-olds and adults, whereas there was no influence of arrow direction on duration judgments in younger children (5 and 8 year olds). This developmental dissociation provides further evidence that the mental timeline is not innate but is acquired during childhood.

However, it remains possible that the younger children in the study by Droit-Volet and Coull (2015) showed no evidence of using a mental timeline for duration simply because they weren’t interpreting or processing the arrow stimuli in the same way as the older children or adults. We previously argued that this was an unlikely explanation because the spatial position indicated by an arrow can be used deliberately by children as young as 3–4 years old to correctly locate a hidden object (Leekham et al., 2010; Jakobsen et al., 2013). Moreover, by 5–6 years of age, the spatial position indicated by an arrow is processed so automatically that it modifies reaction times in spatial orienting tasks (Ristic et al., 2002; Jakobsen et al., 2013; Gregory et al., 2016). Nevertheless, to formally test the possibility that the lack of a mental timeline in 5 year olds was not simply due to their inability to use arrows to indicate spatial position, we conducted a study in which relative spatial position was manipulated either by arrows pointing to the left or right, or by stimuli being physically presented on the left or right side of the computer screen. In addition, to test whether the influence of the mental timeline on perceived duration is due to an underestimation of left-sided stimuli and/or an overestimation of right-sided stimuli, we included control conditions (vertical arrows; central location) to which left- or right-sided stimuli could be separately compared. Based on our previous results using arrow stimuli (Droit-Volet and Coull, 2015), we hypothesized that duration judgments in the youngest children would not be modulated by either arrow direction or stimulus location. If, however, their perceived duration were influenced by stimulus location, this would suggest that our previous results did not reflect the lack of a mental timeline in 5 and 8 year olds. Instead, the arrow stimuli might simply reflect a symbolic representation of time (“time’s arrow”) that has not yet been acquired by these younger children.

## Experiment 1

### Methods

#### Participants

The sample was composed of 58 participants: 19 5-year-olds (mean age = 5.21, *SD* = 0.47, 10 girls), 20 8-year-olds (mean age = 7.48, *SD* = 0.22, 11 girls), and 19 10-year-olds (mean age = 10.18, *SD* = 0.59, 12 girls). Two additional children (one 5-year-old and one 10-year-old) withdrew from the experiment before its completion. Sample sizes were based on prior studies investigating the influence of spatial position on perceived duration (Vallesi et al., 2008; Vicario et al., 2009; Isham et al., 2017). In a sample of three groups of 20 participants, the expected power would be 0.99 for an effect size of 0.15 in our experimental paradigm. All children were recruited from nursery and primary school in Tulle, France and had normal educational backgrounds. Teachers were asked to notify us of any child with learning difficulties. None were identified. Children’s parents and the school headmaster signed a formal agreement to conduct the study. The procedure was validated by the local academic inspection committee of the French National Education Minister.

#### Materials

Children were tested individually in a quiet room in their school. Stimulus presentation and data recording was controlled by E-prime software (Psychology Software Tools). Three different stimuli were used, all composed of the same small rectangle and triangle. The triangle was positioned to the left of the rectangle to create a leftward arrow and to its right to create a rightward arrow. A neutral (vertical) arrow had the triangle positioned either above or below the rectangle randomly across trials. Each stimulus subtended a visual angle of 5°. These three stimuli appeared in one of three different locations on the computer screen – left-lateralized, centered, or right-lateralized – all on the same horizontal plane. In the left and right locations, the center of the stimulus was at 14° eccentricity. In our previous study (Droit-Volet and Coull, 2015), responses were given manually with left and right hands. However, given the increased complexity of this experimental design (simultaneous manipulation of arrow direction and stimulus location) we aimed to facilitate the task for the children by asking them to give their responses verbally. Their responses were then keyed in by the experimenter.

#### Procedure

All children performed a temporal bisection task. In the initial training phase, children were shown the vertical (control) arrow, presented in the center of the screen, for either the short (200 ms) or long (800 ms) standard duration. They were trained to say “short” or “long” when the stimulus had been presented for the short or long standard duration respectively. Each trial started with the word “prêt” (“ready”) displayed on the center of the computer screen. When the participant was ready, the experimenter pressed the spacebar and the stimulus appeared. The participant then responded either “short” or “long,” depending on whether they perceived stimulus duration to be short or long, and the experimenter noted the response. There were eight training trials, with four short and four long durations presented in random order. Previous investigations have shown that eight training trials is sufficient for children in this age-range to understand task instructions (Droit-Volet and Wearden, 2001). Inter-trial intervals were randomized between 0.5 and 1 s. The testing phase immediately followed training. In the testing phase, the stimuli could be leftward, rightward or vertical arrows, and they could be presented on the left, right, or center of the computer screen. For each of these nine experimental conditions, stimulus duration could be 200, 300, 400, 500, 600, 700, or 800 ms. Each testing block thus comprised 63 trials (3 arrows × 3 locations × 7 comparison durations), presented in random order. Participants performed three such blocks, with a short break between blocks, in two separate sessions that were separated by a 15–20 min break. This gave a total of 378 trials (63 trials × 3 blocks × 2 sessions) per participant.

#### Data Analysis

The proportion of “long” responses [*p*(long)] was calculated for each experimental condition. The plot of *p*(long) as a function of the seven stimulus durations constituted a bisection curve. Bisection curves were constructed for each of the nine experimental conditions (3 arrows × 3 locations) for each individual participant. A pseudo-logistic function was fit to the bisection curves using GraphPad Prism 7 software. From the fit of this function to the data, we calculated Bisection Points (BPs) and Weber Ratios (WRs) for each individual participant. The BP is the point of subjective equality and represents the stimulus duration at which participants respond long as often as short, i.e., *p*(long) = 0.50. The lower the BP, the longer the perceived duration. The WR is a measure of temporal sensitivity and is calculated as half the difference between the perceived duration at *p*(long) = 0.75 minus that at *p*(long) = 0.25, divided by the BP. The lower the WR value, the greater the temporal sensitivity and the steeper the psychophysical function.

For each participant, we calculated the average fit across all experimental conditions and excluded any participants with an average fit of *R*^2^ < 0.7 (sixteen 5 year olds, three 8 year olds, and one 10 year old). Data from one additional 8 year-old was also excluded since the BP for one of the experimental conditions was more than double the maximum comparison duration and was therefore considered an outlier. Unfortunately, the fits of the pseudo-logistic function to the data were very poor for the 5 year-olds [mean (± SD) *R*^2^ = 0.46 (± 0.22)]. Due to very low numbers of included 5 year-olds (*n* = 3), we were therefore forced to analyze data from the 8 and 10 year-old groups only (*n* = 16 and 18, respectively).

We first analyzed *p*(long) in a four-way ANOVA with arrow direction (leftward, vertical, rightward), stimulus location (left, center, right) and duration (200, 300, 400, 500, 600, 700, 800 ms) as within-subject factors and age group (8 or 10 years old) as a between-subjects factor. To examine the effect of spatial position on perceived duration and temporal sensitivity, we analyzed BP and WR respectively in two separate three-way ANOVAs, with arrow direction (leftward, vertical, rightward) and stimulus location (left, center, right) as within-subject factors and age group (8 or 10 years old) as a between-subjects factor.

### Results

**FIGURE 1:**
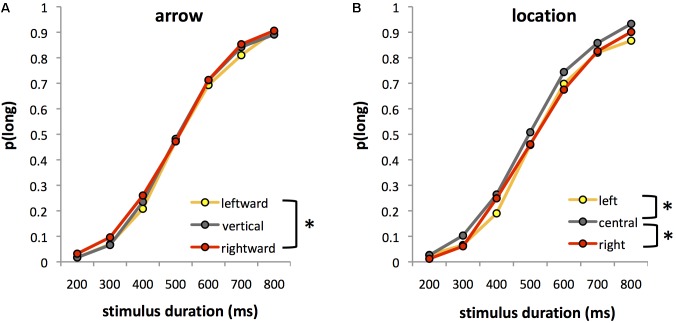
Proportion of trials in which participants judged stimulus duration to be “long” [*p*(long)] for each of the seven comparison durations in Experiment 1. Data are averaged over 8 and 10 year olds, and are plotted as a function of either **(A)** leftward, vertical (control) and rightward arrows, averaged across the three stimulus locations or **(B)** left-lateralized, centralized, or right-lateralized stimuli, averaged across the three arrow directions. Asterisks indicate significant differences between conditions.

**Figure 1** shows the proportion of “long” responses [*p*(long)] in each experimental condition averaged over the 8 and 10 year-old groups. Analysis of *p*(long) revealed the expected significant main effect of stimulus duration, *F*(6,192) = 670.64, *p* < 0.0001, $\eta_{p}^{2}$ = 0.95, with a greater proportion of “long” responses as stimulus duration increased (**Figure 1**).

More interestingly, we found significant main effects of both arrow, *F*(2,64) = 3.04, *p* = 0.055, *$\eta_{p}^{2}$* = 0.09 and location, *F*(2,64) = 20.60, *p* < 0.0001, $\eta_{p}^{2}$ = 0.39. *Post hoc* tests indicated that rightward arrows were more likely to be judged long [mean ± standard error (SE) *p*(long) = 0.48 ± 0.015] than leftward arrows [mean ± SE *p*(long) = 0.45 ± 0.014] (*p* < 0.05, Bonferroni corrected) (**Figure 1A**). The response to vertical arrows [mean ± SE *p*(long) = 0.46 ± 0.016] lay between these two extremes, and was not significantly different to that for either leftward or rightward arrows (both *p* > 0.6). Stimuli presented in the central location were more likely to be judged long [mean ± SE *p*(long) = 0.49 ± 0.015] than those presented on the left (*p* < 0.0001) or right (*p* < 0.0001), with no significant difference between left and right locations [mean ± SE *p*(long) = 0.45 (± 0.015) for both left and right] (**Figure 1B**).

The main effect of age was not significant, *F*(1,32) = 0.37, ns. Moreover, the factor of age did not interact with duration, *F*(6,192) = 0.87, ns, arrow, *F*(2,64) = 1.35, ns nor location, *F*(2,64) = 1.38, ns, indicating that the overall pattern of effect was similar for both age groups.

**FIGURE 2:**
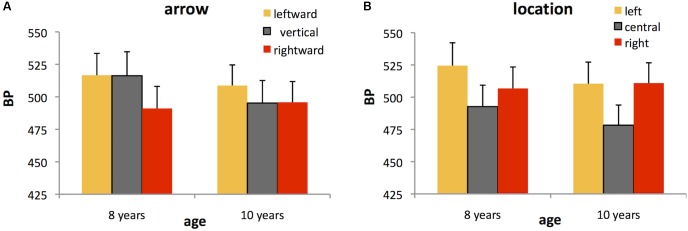
Bisection points (BPs) for each of the spatial conditions in Experiment 1. The higher the BP, the shorter the perceived duration. Data are shown separately for 8 and 10 year olds although there was no significant difference between groups. Data are plotted as a function of either **(A)** leftward, vertical (control), or rightward arrows, averaged across the three stimulus locations or **(B)** left-lateralized, centralized, or right-lateralized stimuli, averaged across the three arrow directions. Error bars represent standard errors.

Analysis of BP confirmed the significant main effects of both arrow, *F*(2,64) = 3.07, *p* = 0.053, ${\text{η}}_{\text{p}}^{2}$ = 0.09, and location, *F*(2,64) = 13.35, *p* < 0.001, ${\text{η}}_{\text{p}}^{2}$ = 0.29, on perceived duration. *Post hoc* tests showed that duration was judged longer for rightward (mean ± SE BP = 493.42 ± 11.67 ms) versus leftward (mean ± SE BP = 512.68 ± 11.55 ms) arrows (*p* < 0.05), with the perceived duration of vertical arrows lying between these two values (mean ± SE BP = 505.74 ± 12.66 ms) (**Figure 2A**). Duration was overestimated for centrally located stimuli (mean ± SE BP = 485.59 ± 11.40 ms) compared to both left- (mean ± SE BP = 517.48 ± 12.16 ms) and right-lateralized stimuli (mean ± SE BP = 508.87 ± 11.44 ms) (both *p* < 0.005) (**Figure 2B**). There was no interaction with age for either arrow, *F*(2,64) = 1.35, ns or location, *F*(2,64) = 1.38, ns, indicating that the effects of arrow and location on perceived duration were similar for both age groups.

In terms of temporal sensitivity, there were no significant effects of either arrow, *F*(2,64) = 0.07, or location, *F*(2,64) = 2.37, on WR, nor an interaction of either of these factors with age (all *p* > 0.05). We found only a main effect of age, *F*(1,32) = 4.68, *p* < 0.05, ${\text{η}}_{\text{p}}^{2}$ = 0.13, indicating that 8 year olds had wider curves (mean ± SE WR = 0.23 ± 0.015), and therefore poorer temporal sensitivity than 10 year olds (mean ± SE WR = 0.19 ± 0.014).

### Discussion

These results indicate that stimulus location significantly modified perceived duration in both age groups. However, the pattern of effect was not in the predicted direction. Based on existing literature (Vicario et al., 2008; Bonato et al., 2012), we hypothesized that right-lateralized stimuli would be overestimated compared to left-lateralized ones. However, we found that centrally located stimuli were judged longer than either left- or right-sided stimuli, with no difference between the two lateralized locations. One possible mechanism for this unexpected finding is that the spatial position indicated by arrows and stimulus location interfered with one another, leading participants to focus on the trained and ‘spatially neutral’ central location. Since the perceived duration of attended stimuli is longer than that of unattended stimuli (Brown, 2008), focusing attention on the central location might have led participants to overestimate the duration of stimuli presented there. In any case, we found no evidence for differential effects of left- versus right-lateralized stimuli on perceived duration in either of the age groups.

Our data do, however, confirm and extend previous results (Droit-Volet and Coull, 2015) that arrow direction had a significant impact on perceived duration in children older than 7 years old, with rightward-facing stimuli being judged longer than leftward stimuli. Nevertheless, effect sizes were rather small and should be interpreted with caution. Small effect size could be due to the fact that in the current study we manipulated both arrow direction *and* stimulus location simultaneously, whereas we manipulated only arrow direction in our previous study (Droit-Volet and Coull, 2015). The interaction between potentially conflicting representations of spatial position in the current study may have diluted the influence of the mental timeline on perceived duration. Indeed, simultaneous manipulation of arrow direction and stimulus location severely impaired performance in the youngest children. Unfortunately, data from only three of the nineteen 5 year olds were orderly enough to be fit by a psychometric curve, and so this age group had to be excluded from analyses. The increase in combinatorial possibilities of arrow direction and stimulus location (nine experimental conditions presented in randomized order) likely confused these young children, who responded rather randomly. Indeed, Iarocci et al. (2009) also reported that the combined presentation of arrows and lateralized locations impaired performance on a spatial cueing paradigm in 5 year olds, but not 7 or 9 year olds. Although 5 year olds were able to successfully use predictive arrows to quickly detect a lateralized target, these performance benefits were lost when the arrow cue was immediately preceded by presentation of an uninformative, but salient, peripheral stimulus.

Importantly, if 5 year-olds were not performing our duration estimation task as required, we could not assess the effects of stimulus location on perceived duration in this age group. In our previous study (Droit-Volet and Coull, 2015), we manipulated a single spatial factor: arrow direction. In the current study, we manipulated both arrow direction and stimulus location, which proved too complicated for the 5 year olds. Therefore, to try and facilitate the task for young children, and so allow us to measure the influence of the mental timeline in 5 year olds, we developed a simpler paradigm in which we modulated stimulus location only. In this follow-up experiment, we compared the perceived stimulus duration of a circle that appeared in either left, right, or central locations. This is equivalent to the task used in Experiment 3 of Vicario et al. (2008), although we used a temporal bisection task rather than temporal discrimination. We used the same short and long anchor durations as Experiment 1 (200 ms and 800 ms) but, to make the task shorter, included just five comparison durations rather than seven.

## Experiment 2

### Methods

#### Participants

The sample was composed of 44 participants: 20 5–6 year-olds (mean age = 5.62 years, *SD* = 0.29, range: 5.08–6.08 years; 13 females) and 24 adults (mean age = 20.13 years, *SD* = 3.71, range: 18–33 years; all female). Although there was a discrepancy in the gender balance for children (65% female) and adults (100% females), Espinosa-Fernández et al. (2003) have reported that there are no gender differences in estimating duration in the seconds range for participants under 60 years old. All children were recruited from nursery schools in Clermont-Ferrand, France and had normal educational backgrounds. Teachers were asked to notify us whether any child had learning difficulties. None were identified. Children’s parents and the school headmaster signed a formal agreement to conduct the study. The procedure was validated by the local academic inspection committee of the French National Education Minister.

#### Materials

Children and adults were tested in a quiet room in school or University. Stimulus presentation and data recording was controlled by E-prime software (Psychology Software Tools). The stimulus was a black circle (3° visual angle), which appeared in one of three locations on the computer screen – left-lateralized, centered, or right-lateralized (eccentricity of left and right locations was 14°). To facilitate the task for the children, they gave their responses orally (“short” or “long”), which were then keyed in by the experimenter. Adults gave responses by pressing the upward or downward arrow key of the computer keyboard with their right index finger. Eikmeier et al. (2015b) have previously shown that spatial location influences the processing of temporal words in similar ways whether participants respond vocally or manually. For adults, the upward and downward keys had “short” or “long” stickers placed upon them, with the association between up/down key and short/long response counterbalanced across participants. We chose response keys positioned in the vertical axis (up/down), rather than the horizontal one (left/right), to avoid possible interference effects between stimulus position and side of response (Simon, 1969).

#### Procedure

All participants performed a temporal bisection task. In the initial training phase, participants were first shown the stimulus in the central location for either the short (200 ms) or long (800 ms) standard duration, four times each (eight trials). Each trial started with the word “prêt” (“ready”) displayed on the center of the computer screen. When the participant was ready, the spacebar was pressed (either by the experimenter for the children or by the participant for the adults) and the stimulus appeared. The participant gave either a “short” or “long” response, depending on whether they perceived stimulus duration to be short or long. There were eight training trials, with four short and four long durations presented in random order. The testing phase immediately followed training. In the testing phase, the stimulus was presented on the left, right, or center of the computer screen. For each of these three experimental conditions, stimulus duration could be 200, 350, 500, 650, or 800 ms (15 trial-types). Participants performed a total of 120 trials (eight repetitions of each of the 15 trial-types).

#### Data Analysis

The proportion of “long” responses [*p*(long)] was calculated for each experimental condition. Bisection curves were constructed for each of the three stimulus locations for each individual participant. A pseudo-logistic function was fit to the bisection curves using GraphPad Prism 7 software. From the fit of this function to the data, we calculated BPs and WRs for each individual participant. Fits were calculated separately for each of the three locations. Data from only one child (BP more than double the maximum stimulus duration) and one adult (BP could not be calculated due a very poor fit) had to be excluded from the analyses. The improved fit for 5–6 year olds’ data in this experiment compared to Experiment 1 indicates the utility of manipulating only a single experimental factor at a time when measuring duration estimates in young children.

We first analyzed *p*(long) in a three-way ANOVA with stimulus location (left, center, right) and duration (200, 350, 500, 650, or 800 ms) as within-subject factors and age group (children, adults) as a between-subjects factor. To examine the effect of location on perceived duration and temporal sensitivity, we analyzed BP and WR in two separate two-way ANOVAs with stimulus location (left, center, right) as a within-subject factor and age group (children, adults) as a between-subjects factor.

## Results

**FIGURE 3:**
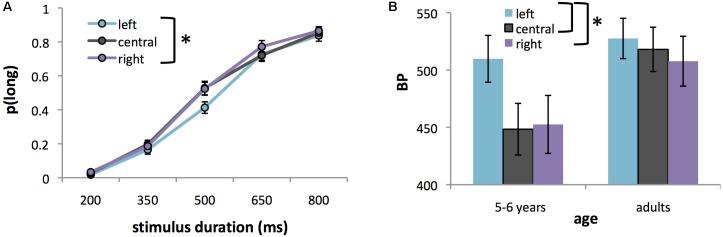
**(A)** Proportion of long responses [*p*(long)], averaged over both age groups in Experiment 2, for stimuli appearing on the left, center, or right of the computer screen plotted against stimulus duration. **(B)** BPs for left-lateralized, centralized, or right-lateralized stimuli in 5–6 year olds and adults of Experiment 2. The higher the BP, the shorter the perceived duration. Error bars represent standard errors. Asterisks indicate significant differences between conditions.

An ANOVA of *p*(long) revealed the expected significant main effect of stimulus duration, *F*(4,168) = 355.84, *p* < 0.0001, ${\text{η}}_{\text{p}}^{2}$ = 0.89 with *p*(long) increasing as a function of stimulus duration (**Figure 3A**). More interestingly, we also found a significant main effect of location, *F*(2,84) = 5.10, *p* < 0.01, ${\text{η}}_{\text{p}}^{2}$ = 0.11. *Post hoc* tests revealed that duration was judged longer for stimuli presented on the right [mean ± SE *p*(long) = 0.48 ± 0.022] versus left [mean ± SE *p*(long) = 0.43 ± 0.020] of the screen (*p* = 0.005). There was no significant difference between centrally located stimuli [mean *p*(long) = 0.47 ± 0.020] and either left- or right-lateralized stimuli (*p* > 0.05). The main effect of location was qualified by a significant location × duration, *F*(8,336) = 2.12, *p* < 0.05, ${\text{η}}_{\text{p}}^{2}$ = 0.05) interaction (**Figure 3A**), with the effect being significant for the 500 ms duration only (i.e., midway between the short and long anchor durations). There was no significant main effect of age, *F*(1,42) = 0.93, ns, nor an age × location interaction, *F*(2,84) = 2.38, ns. The only effect of age was a significant age × duration interaction, *F*(4,168) = 8.28, *p* < 0.0001, ${\text{η}}_{\text{p}}^{2}$ = 0.17, with *post hoc* tests revealing that 5–6 year olds overestimated the two shortest comparison durations [mean *p*(long) = 0.04 and 0.29 for the 200- and 350-ms durations respectively, *p* < 0.005] as compared to adults [mean *p*(long) = 0.01 and 0.08 for the 200- and 350-ms durations respectively, *p* < 0.0001].

Analysis of BP confirmed a significant main effect of location, *F*(2,80) = 4.87, *p* = 0.01, ${\text{η}}_{\text{p}}^{2}$ = 0.11 on perceived duration. *Post hoc* tests showed that duration was judged significantly shorter for left-lateralized stimuli (mean ± SE BP = 523.69 ± 16.75 ms) compared to right-lateralized (mean BP ± SE = 485.97 ± 16.82 ms) or centrally located ones (mean ± SE BP = 480.26 ± 14.16 ms) (all *p* < 0.05, Bonferroni corrected). The age by location interaction was not significant, *F*(2,80) = 2.51, ns, although there was a trend (*p* = 0.088, ${\text{η}}_{\text{p}}^{2}$ = 0.06) for the effect of location to be stronger in children than in adults (**Figure 3B**).

As in Experiment 1, there were no significant effects of location on WR [*F*(2,80) = 1.83, ns] nor a location × age interaction, *F*(2,80) = 0.10, ns, although the main effect of age was significant, *F*(1,38) = 14.00, *p* < 0.001, ${\text{η}}_{\text{p}}^{2}$ = 0.31), indicating that children (mean ± SE WR = 0.29 ± 0.024) had a wider curve (i.e., lower temporal sensitivity) than adults (mean ± SE WR = 0.16 ± 0.022).

### Discussion

In Experiment 1, the poor performance of 5 year-olds made it impossible to test the influence of spatial position on perceived duration. We therefore designed a simpler paradigm for Experiment 2, in which a non-spatial stimulus (filled black circle) was presented in one of three locations. Using this simpler paradigm, we found significant differences in perceived duration of left- versus right-lateralized stimuli even in 5–6 year-olds. Stimuli appearing on the left of the screen were perceived as shorter than those appearing on the right. There were no effects of stimulus position on temporal sensitivity, which is in line with results of our previous study (Droit-Volet and Coull, 2015), and of Experiment 1. The mental timeline therefore influences the accuracy, but not precision, of subjectively perceived duration. These data confirm the results of Vicario et al. (2008) in adults and, moreover, extend this finding to children as young as 5–6 years old.

Indeed, there was a trend for the effect to be even stronger in children than in adults. Although this effect was not statistically significant, the pattern of effect might be due to the fact that children gave responses verbally while adults gave responses manually. Although we took care to control for possible interference between the side of stimulus presentation and manual response-mode (see section “Methods”), it’s possible that adults might have shown even stronger effects of stimulus location if they had given their responses verbally, for which no response interference is possible. Alternatively, the trend for the effect to be stronger in children than adults might be because children are more susceptible than adults to the influence of non-temporal contextual factors, such as sensory modality (Droit-Volet and Hallez, 2018), stimulus magnitude (Droit-Volet et al., 2008), visual salience (Charras et al., 2017) or, in this case, stimulus location.

The significant difference in the perceived duration of left-lateralized versus centrally located stimuli indicates that the difference between left and right-lateralized stimuli might be more accurately interpreted as an underestimation of left-sided stimuli, rather than an overestimation of right-sided ones. This finding replicates the pattern observed by Vicario et al. (2009) in a cross-modal timing study of adults. In their study, a visual stimulus was judged to have a shorter duration when auditory distractors were presented to the left ear, as compared to distractors in the either the right, or both, ears. By contrast, the temporal effect of distractors presented to the right ear was no different to that of bilateral distractors. Most studies of the mental timeline directly compare left and right lateralized stimuli to one another, making it impossible to conclude whether effects are due to an overestimation of right-sided stimuli and/or an underestimation of left-sided ones. It’s unclear why left-lateralized stimuli might influence duration processing more than right-sided ones, but our results indicate that future studies of the mental timeline should ideally include a spatially neutral condition to determine whether this pattern is replicable. One speculative explanation is that the right hemisphere specialization for spatial attention (Corbetta and Shulman, 2002) preferentially weights processing of stimuli presented in the left visual field, meaning that the effects of left-lateralized stimuli on duration estimation contribute more to the overall pattern of effect than those of right-lateralized ones.

## General Discussion

We conducted two experiments to pinpoint the age at which spatial position begins to influence children’s temporal judgments, which would indicate the use of a mental timeline to represent duration. Children (aged 5–6, 8, or 10 years old) and adults performed temporal bisection tasks in which relative spatial position (left/right) was manipulated by either arrow direction (Experiment 1) and/or lateralized stimulus location (Experiments 1 and 2). An influence of relative spatial position on perceived duration was hypothesized to signify a spatial representation of time, consistent with the use of a mental timeline. Our results confirmed previous findings (Droit-Volet and Coull, 2015) that arrow direction influenced subjective estimates of duration in older children. In the current study, we found that duration of leftward facing arrows was perceived to be shorter than that of rightward facing ones in both 8 and 10 year olds. Contrary to our hypothesis, however, stimulus location modulated perceived duration in children as young as 5–6 years old, with duration of left-lateralized stimuli being judged shorter than that of either right-lateralized or central stimuli. These data are consistent with the use of a mental timeline for stimulus duration from the age of 5–6 years old, with short duration being represented on the left side of space and long duration on the right.

### Children Have a Mental Timeline for Duration

In a prior study, we had concluded that the influence of arrow direction on duration judgments in older, but not younger, children reflected the acquisition of a mental timeline around the age of 8–10 years old (Droit-Volet and Coull, 2015). We took this developmental dissociation as further proof that the mental timeline is a culturally acquired representation of duration (Bonato et al., 2012; Núñez and Cooperrider, 2013; Winter et al., 2015; Magnani and Musetti, 2017). Nevertheless, by representing spatial location physically (left/right side of the screen) rather than symbolically (left/right arrow), we have now found evidence that children as young as 5–6 years old may also represent duration along a left–right axis. By age 5, children in Western cultures have already begun to acquire the habit of reading from left to right, and are likely to have number lines going from left to right pinned to the walls of their classrooms. Because words on the left are read before those on the right, a conceptual association is created between the order of events in time and relative spatial position. Such cultural conventions therefore influence the way in which we conceptualize the notion of temporal order: events that happen first are represented on the left whereas those that happen later are represented on the right (Fuhrman and Boroditsky, 2010; Hendricks and Boroditsky, 2015). Our data, along with many previous findings (e.g., Vallesi et al., 2008; Vicario et al., 2008), suggest that these cultural conventions also influence our notion of *duration*: events of short duration are represented on the left and longer ones on the right. The mechanism that would create an association between duration and spatial position is perhaps less intuitive than that between temporal order and spatial position. But one obvious possibility is that as we read from left to right, time elapses. Therefore, reading duration lengthens as we move from left to right on the page, creating an association between duration and position. Of course, this association would have to be reset every time we began again on the next line, making it less robust and perhaps explaining why there is less experimental evidence for a mental timeline for duration than there is for a mental timeline for temporal order (Bonato et al., 2012). Nevertheless, our data indicate that a mental timeline for duration appears to be in operation from the age of 5 years old.

### Children’s Perception of Duration Is Influenced by Spatial Position as Well as Spatial Magnitude

Our findings complement previous developmental studies (Casasanto et al., 2010; Bottini and Casasanto, 2013; Charras et al., 2017) showing that 5 year olds represent duration in terms of spatial magnitude (i.e., size or distance). Young children are therefore able to conceptualize the duration of an event in terms of either spatial magnitude (short duration is equivalent to small size) or spatial position (short duration is equivalent to the left side of space). Importantly, these two distinct ways of representing duration in spatial terms – magnitude or position – are not mutually exclusive and may simply represent two different mechanisms for rendering the abstract notion of time a little more tangible (Núñez and Cooperrider, 2013; Winter et al., 2015). Additional experiments in younger children are now needed to confirm whether the spatial representation of duration develops first in terms of spatial magnitude before then being influenced, through cultural habits, by spatial position.

### Developmental Dissociation in the Effects of Arrows or Location on Perceived Duration

Crucially, the youngest children only showed evidence of a timeline when spatial position was represented physically by the location of the stimulus on the screen, not when represented symbolically by an arrow (Droit-Volet and Coull, 2015). While the physical location of a stimulus captures the focus of spatial attention automatically, or “exogenously,” arrows direct spatial attention in a more voluntary, or “endogenous” way. Although there is evidence that children in this age range orient attention automatically to the spatial location indicated by an arrow (Ristic et al., 2002; Jakobsen et al., 2013), it seems that this attentional mechanism is not yet strong enough to induce a knock-on effect on the perception of time. Indeed, the spatial attentional mechanisms induced by arrow stimuli mature later than those induced by the physical location of the stimulus (Brodeur and Enns, 1997; Ristic and Kingstone, 2009). Therefore, arrows might simply be an ineffective way of measuring how the locus of spatial attention modulates perceived duration in these younger children. Alternatively, arrows might be processed as a symbolic representation of “time’s arrow” that is acquired only later in development, thus explaining why perceived duration is modulated by arrow direction only from the age of 8 years old (Droit-Volet and Coull, 2015).

### Experimental Parameters Affect Whether Spatial Position Modulates Perceived Duration

Finally, our pattern of findings highlight that specific experimental parameters will dictate whether or not we are likely to find evidence of a mental timeline for duration. For example, we found a significant difference in the perceived duration of stimuli appearing on the left versus right of the screen in Experiment 2, in which stimuli were simple black dots, but not in Experiment 1, in which stimuli were arrows. Task demands are known to modulate the strength of influence of the mental timeline (Rolke et al., 2013). It’s therefore possible that the direction in which the focus of spatial attention was oriented endogenously by arrows in Experiment 1 interfered with the way in which attention was oriented exogenously by stimulus location, diluting any effects of lateralized spatial location on perceived duration. The interaction between endogenous and exogenous attentional mechanisms is immature before the age of 6 years old (Iarocci et al., 2009; Ristic and Kingstone, 2009) and, indeed, the factorial combination of arrows and locations proved too much for 5 year olds, whose performance on the task broke down completely. Even in the older children, we found no evidence for differential effects of left- versus right-sided stimuli on perceived duration. Instead, in Experiment 1, perceived duration was longer for stimuli appearing in the *center* of the screen rather than either the left or the right. Since the duration of attended stimuli are judged longer than non-attended ones (Brown, 2008), this unexpected result might be due to the fact that in this complex experiment, children focused their attention on the central location, inadvertently lengthening the perceived duration of stimuli appearing there. We acknowledge this is a *post hoc* explanation for an unexpected finding. Nevertheless, it’s important to stress that the simplification of the task in Experiment 2 unveiled the effects of location on perceived duration, unencumbered by the potentially interfering effects of arrow direction.

Even though the combination of arrows and locations in Experiment 1 modified the expected effects of location on duration, it did not alter the effects of arrow direction on duration. In fact, we not only confirmed that 10 year olds perceive leftward arrows as having a shorter duration than rightward ones (Droit-Volet and Coull, 2015) we also found this effect in 8 year olds. This suggests that, at least in older children and adults, effects of symbolic arrows are potentially stronger than effects of physical location. Although this seems counterintuitive, one possible explanation for this comes from the very recent work of Isham et al. (2017). In this study, stimulus laterality (left/right) and stimulus eccentricity (central/peripheral) were manipulated conjointly. Although left-lateralized stimuli were estimated to have shorter duration than right-lateralized ones, this was true only when stimuli were presented within 3 degrees of central fixation. In fact, when stimuli were presented in more peripheral locations (8 degrees of eccentricity), these findings were reversed. Therefore, the influence of the mental timeline on duration depends upon the degree of lateral spatial displacement from the midline (Isham et al., 2017). In Experiment 1, our centrally located arrowhead was 2 degrees from the midline whereas arrowheads in the peripheral locations were 11 or 16 degrees from the midline. According to the results of Isham et al. (2017), the underestimation of left-lateralized stimuli should be more evident when relative position is manipulated by arrow direction (2 degrees) rather than by its peripheral location on the screen (11/16 degrees). This is precisely what we found. Nevertheless, Isham et al. (2017) also showed that in the periphery, left-sided stimuli were *over*-estimated. This contradicts our results in Experiment 2, which showed underestimation of left-lateralized stimuli. It may be that when location and eccentricity (Isham et al., 2017) are manipulated together, or indeed location and arrow direction (Experiment 1), the effects of the mental timeline on duration are less clear. In addition, given that the arrow stimuli in our study could be presented on the left, center or right of the screen, the crucial factor may not be eccentricity from the midline of the screen, but eccentricity from a point of reference such as the midline of the arrow stimulus itself. Further experiments would obviously be required to test this *post hoc* explanation.

## Conclusion

We found that spatial position influences perceived duration in children as young as 5–6 years old, indicating the use of a mental timeline to represent duration from a relatively young age. Nevertheless, this effect was found in these younger children only when spatial position was manipulated by varying the left/right location of the stimulus on the screen. If spatial position was conveyed in a more symbolic manner by leftward/rightward facing arrows, then the effect of position on perceived duration was found only in children aged 8 years and older (Droit-Volet and Coull, 2015). This age-related dissociation may reflect development of the conceptual understanding that “time’s arrow” flows from left to right around the age of 8 years olds. Alternatively, it could reflect distinct developmental trajectories of automatic versus voluntary attentional mechanisms, which are differentially engaged when spatial position is manipulated by the physical location of a stimulus or the direction of an arrowhead. This explanation highlights the importance of taking attentional ability into account when interpreting results of duration judgment tasks (Droit-Volet, 2016; Hallez and Droit-Volet, 2017). Unfortunately, we did not use neuropsychological tests to assess childrens’ memory or attentional function in the present study. Future investigations should examine how underlying cognitive capacity affects the influence of spatial context on perceived duration.

Furthermore, it would be extremely informative to repeat this experiment in a group of children who read from right-to-left, the prediction being that left-lateralized stimuli would now be overestimated compared to right-lateralized ones. Importantly, such an experiment might help clarify why, in the current experiment, we found an underestimation of left-sided stimuli rather than an overestimation of right-sided ones. If our pattern of effect was indeed due to 5 year olds’ bias to process left-lateralized stimuli then, as compared to central stimuli, there should be a disproportionate overestimation of left-sided stimuli in the right-to-left reading population, as opposed to an underestimation of right-sided ones.

Finally, it is important to remember that even though the spatialization of time might provide a useful heuristic for communication, it is not the only way that time can be represented. Kranjec and Chatterjee (2010) note that spatial metaphors for time (e.g., look *forward* to the party) appear later in language development than purely temporal words (e.g., the party is *tomorrow*), suggesting that time could be represented independently of space. They suggest, as an alternative framework, that time might be represented in sensorimotor networks of the brain. This hypothesis is supported by converging evidence from both the neuroimaging and developmental domains (Coull and Droit-Volet, unpublished). For example, structures of the brain typically associated with motor function, such as Supplementary Motor Area or basal ganglia, are activated by purely perceptual timing tasks (Wiener et al., 2010; Coull et al., 2011). In parallel, young children appear better able to represent time when it is coupled to a motor act (Droit-Volet, 1998; Droit-Volet and Rattat, 1999). Untangling the distinct, and overlapping, contributions of spatial and sensorimotor experience to our understanding of time is an important challenge for future research.

## Data Availability

The raw data supporting the conclusions of this manuscript will be made available by the authors, without undue reservation, to any qualified researcher.

## Author Contributions

JC, KAJ, and SD-V conceived the experiments and wrote the paper. SD-V acquired the data. JC and SD-V analyzed the data.

## Conflict of Interest Statement

The authors declare that the research was conducted in the absence of any commercial or financial relationships that could be construed as a potential conflict of interest.
